# Nuclear Entry of Hepatitis B Virus Capsids Involves Disintegration to Protein Dimers followed by Nuclear Reassociation to Capsids

**DOI:** 10.1371/journal.ppat.1000563

**Published:** 2009-08-28

**Authors:** Birgit Rabe, Mildred Delaleau, Andreas Bischof, Michael Foss, Irina Sominskaya, Paul Pumpens, Christian Cazenave, Michel Castroviejo, Michael Kann

**Affiliations:** 1 Institute of Virology, Justus Liebig University, Giessen, Germany; 2 UMR 5234 CNRS-University Bordeaux 2 MCMP, Bordeaux, France; 3 Latvian Biomedical Research and Study Centre, Riga, Latvia; Harvard Medical School, United States of America

## Abstract

Assembly and disassembly of viral capsids are essential steps in the viral life cycle. Studies on their kinetics are mostly performed *in vitro*, allowing application of biochemical, biophysical and visualizing techniques. *In vivo* kinetics are poorly understood and the transferability of the *in vitro* models to the cellular environment remains speculative. We analyzed capsid disassembly of the hepatitis B virus in digitonin-permeabilized cells which support nuclear capsid entry and subsequent genome release. Using gradient centrifugation, size exclusion chromatography and immune fluorescence microscopy of digitonin-permeabilized cells, we showed that capsids open and close reversibly. In the absence of RNA, capsid re-assembly slows down; the capsids remain disintegrated and enter the nucleus as protein dimers or irregular polymers. Upon the presence of cellular RNA, capsids re-assemble in the nucleus. We conclude that reversible genome release from hepatitis B virus capsids is a unique strategy different from that of other viruses, which employs irreversible capsid destruction for genome release. The results allowed us to propose a model of HBV genome release in which the unique environment of the nuclear pore favors HBV capsid disassembly reaction, while both cytoplasm and nucleus favor capsid assembly.

## Author Summary

Viral capsids facilitate protection of the enclosed viral genome and participate in the intracellular transport of the genome. At the site of replication capsids have to release the genome, but after replication new capsids have to be assembled for encapsidation of the progeny genomes. Detailed data on stability of capsids and kinetics of their formation and dissociation are obtained for several viruses *in vitro*, allowing biophysical or electron microscopical techniques. These approaches, however, do not consider the impact of cellular interaction partners. Using digitonin-permeabilized cells which support hepadnaviral genome release actively, we analyzed the disassembly kinetic of the hepatitis B virus (HBV) capsid. Using different analytical methods we found that HBV capsids disintegrate to protein dimers which reassemble to capsids inside the nucleus. The study provides a link between *in vitro* and *in vivo* data showing that HBV uses a unique strategy. We propose a model in which the unique environment of the nuclear pore favors the disassembly reaction, while both cytoplasm and nucleus favor assembly.

## Introduction

Viral capsids facilitate multiple functions in the viral life cycle. Outside the cell, they protect the enclosed viral genome against nucleases, and in case of non-enveloped viruses they mediate attachment and entry. For both enveloped and non-enveloped viruses, they carry the viral genome to the site of replication where they have to release the genome in order to allow access of transcription and/or replication factors. After replication new capsids have to be assembled for encapsidation of the progeny genomes and subsequent release of mature virions. Capsids are assigned to be metastable: early in infection they have to open, later they have to assemble and close. Most data on stability of capsids and kinetics of their formation and dissociation are obtained *in vitro* allowing analysis by biophysical or electron microscopical techniques (e.g. [1]–[5])

*In vivo* data on capsid disintegration are rare despite of their importance for genome release and viability of infection. Two different examples are Adenovirus 5 (Ad-5) capsids and the capsid of Herpes simplex virus-1 (HSV-1). Ad-5 capsids disintegrate to penton and hexon subunits after modification upon endocytosis [6],[7] for genome release. Capsids of HSV-1 in contrast remain stable after release of one penton [8] which allows the injection of the viral DNA from that opening [9].

Intensive studies on capsid assembly were performed *in vitro* on capsids of the medically important Hepatitis B virus (HBV) [1],[5],[10],[11]. HBV infection is endemic in large parts of the world and ∼350 Mio people are chronically infected, accounting for 1 million deaths per year. HBV is an enveloped virus with an icosahedral capsid that is composed of 240 or 180 copies of a single protein species called core protein [12]. Within the oxidizing environment outside the cell the two core protein subunits of a dimer become linked by three disulfide bonds (Cys 48, 61 and 183 [13],[14]). The capsid encloses the relaxed circular viral DNA (rcDNA) [15], which is covalently attached to the viral polymerase [16].

HBV cell entry is the limiting step that prevents infection of most cell cultures but it can be by-passed by lipofection of hepatoma cells with virion-derived capsids. Using this artificial mode of capsid entry, HBV production reaches *in vivo*-like efficiency [17]. This suggests that the capsids either do not become modified during natural entry or that lipofection changes their structure in the same way. Complex interactions with the cellular transport machinery mediate capsid translocation to the nuclear periphery, passage through the NPC and liberation of the viral DNA [17]–[19].

Transport and genome release must be highly efficient and well-coordinated, because ∼80% of virions are infection-competent *in vivo* [20]. Within the nucleus, viral DNA is converted by cellular repair mechanisms to a covalently closed circular form (cccDNA), which is the template for viral mRNA synthesis including the RNA pregenome. Interaction of the RNA pregenome with the viral polymerase facilitates encapsidation into the viral capsid [21]. The polymerase retrotranscribes RNA pregenome into the rcDNA, but this occurs only within the capsids. This genome maturation requires multiple phosphorylation steps within the C terminus of the core protein [22],[23]. Mature capsids (Mat-C) can either be enveloped by the viral surface proteins in order to form virions or they can be transported through the NPC causing amplification of nuclear viral DNA.

Liver histology of HBV-infected individuals shows massive intranuclear capsid accumulation. However, the number of intranuclear viral genomes is low. Thus, it is believed that unassembled core protein pass the NPC and assembles intranuclear [24].

HBV capsids exhibit predominantly a T = 4 symmetry with a diameter of ∼36 nm. Core protein assembly is independent of eukaryotic host proteins as it occurs also upon core protein expression in *E. coli* resulting in “recombinant” capsids (rC). In contrast to natural capsids, rC are unphosphorylated and contain *E. coli* RNA [25]. In addition, they exhibit one unnatural disulfide bond linking the C terminal Cys (C183) of one core protein with a C183 of a neighboring dimer [26].

Structure of the first 143 aa of core proteins in rC has been obtained by X ray crystallography with a resolution of 3.3 Å [27]. The core monomer comprises two long α-helices with a hairpin structure. Association of two hairpins from two monomers forms a spike which protrudes from the particle surface. The connecting loop is exposed on the spike tip and comprises the immune dominant c/e1 B cell epitope, so that most antibodies are conformation-dependent. While the first 143 aa of the core protein are well structured [12], the C terminus is flexible: whereas the C terminus is localized within capsid lumen in *E. coli*-expressed capsids [28], it is exposed to the exterior in viral mature capsids [19]. The latter observation may however indicate that capsids dynamics increase with genome maturation.

*In vitro* association kinetics, performed on *E. coli*-expressed, C terminally truncated core proteins (aa 149), showed that capsid formation starts with core protein dimers. It is thought that the dimers trimerize and the resulting hexamer nucleates capsid assembly without accumulating further distinct populations of capsid subassemblies [10],[29]. According to the laws of thermodynamics, disassembly could just be the inverse reaction because the attractive forces between the subunits are weak, allowing them to transiently dissociate and re-associate (capsid breathing) [1],[5] in a way similar to that observed for polio-, flockhouse- and rhinoviruses [2]–[4]. In fact, recent *in vitro* evaluations showed that chaotropic agents as urea cause disassembly down to core protein dimers without distinguishable capsid subassemblies [30]. Several differences of these *in vitro* studies with the *in vivo* situation deserve attention: the C terminus, which interacts with encapsidated nucleic acids [31] and comprises the phosphorylation sites, was deleted in these studies; the capsids contained neither RNA nor DNA nor the polymerase. Moreover, host factors explaining the highly time- and site-coordinated HBV genome release were not present.

Accounting for the poor knowledge on *in vivo* disassembly and the medical importance of HBV, we evaluated the fate of HBV capsids within the cell. As no efficient infection system exists, we used digitonin-permeabilized cells that promote genome liberation into the nucleus. In order to distinguish reliably between input capsids and products of disassembly and re-assembly, we determined antibody binding properties, density, and size of authentic and recombinant capsids, and compared these particles with the products of nuclear import.

## Results

### Overview

We characterized ^32^P labelled mature DNA-filled capsids from cell culture and RNA-filled capsids from *E. coli* as reagents. These capsids exhibited different densities upon Nycodenz gradient centrifugation. DNA-filled capsids undergo a transition to a density that resembled the RNA-filled state that correlates with nuclear entry. We demonstrated that *E. coli* expressed capsids dissociate and reassociate. Using the assembly/disassembly states we characterized two anti core protein antibodies and found different specificities for assembly states. These characteristics allowed us to analyse the intracellular fates of DNA-filled capsids by immune fluorescence microscopy.

### Characterization of capsids and core proteins

**Figure 1:**
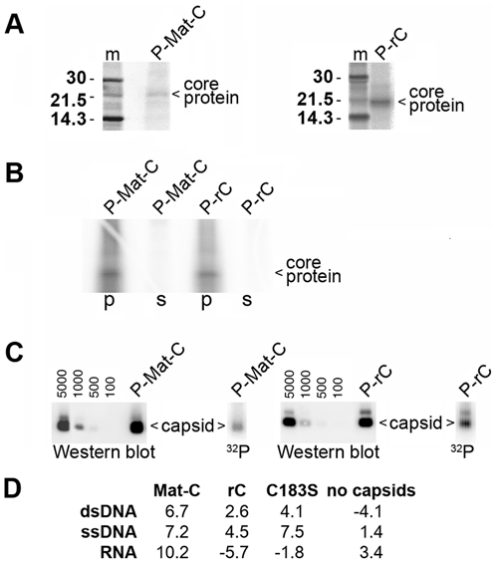
Analysis of ^32^P labelled capsids. A. Labelling of virion-derived capsids (P-Mat-C) using the endogenous protein kinase (left) and *E. coli*-derived capsids (rC) by *in vitro* phosphorylation (P-rC, right) using SDS PAGE. m: ^14^C marker, labelled virion-derived capsids (P-Mat-C), labelled *E. coli*-expressed capsids (P-rC). The labelling resulted in a single 21.5 kDa band characteristic for the core protein. B. Immune precipitation of both capsids by DAKO AB. p: precipitate, s: supernatant. Both capsids were completely precipitated. The result shows that no other radiolabelled 21.5 kDa protein contaminated the preparations. C. Separation of P-Mat-C (left) and P-rC (right) on native agarose gels followed by Western blot using DAKO Ab or phosphoimaging (^32^P). The numbers show a geometrical dilution of rC in pg, which was used for quantification. The figures show that the labelled core proteins were assembled to capsids. D. Nuclease activity in the capsid preparations. ^32^P-labelled nucleic acids were incubated for 2 h at 37°C with 50 ng of capsids. The numbers show the % of hydrolysis based on duplicates and depicts that no significant DNase or RNase activity was contaminating the preparations.

Phosphorylation of both HBV virion-derived (Mat-C) and *E. coli*-derived (rC) capsid species resulted in specific labelling of the core proteins (Fig. 1A). In order to exclude that contaminant proteins accounted for the radiolabelled 21.5 kDa band we performed an immune precipitation using a polyclonal anti HBV capsid antibody (DAKO Ab). This antibody does not react with denatured core protein or core protein aggregates generated by acidification [18]. Fig. 1B showed that the DAKO Ab completely precipitated the 21.5 kDa band. This finding confirmed the identity of the band as core protein and showed that no other ^32^P-labelled protein co-migrated. It further indicated that all core proteins exhibited their proper conformation after phosphorylation. To analyze whether the core proteins were assembled to capsids we performed a native agarose gel electrophoresis of (Fig. 1C). No disintegrated capsids or aggregated core proteins exhibiting slower and diffuse migration were observed [18]. Phosphorylated rC (P-rC) caused a minor, slower migrating band. The additional band is characteristic for *E. coli*-expressed capsids. It is assumed that the minor band represent two capsids linked by RNA, as RNase A-treatment reduces the slower migrating band. The presence of this band thus indicates that the trapped RNA was not degraded upon *in vitro* phosphorylation. Both capsid species reacted with the DAKO Ab confirming their exposure of core protein epitopes.

We next analyzed possible contaminations with nucleases in the capsid preparations. We used ^32^P labelled dsDNA, ssDNA and RNA and determined the hydrolysis after 2 h incubation at 37°C (Fig. 1D). We observed reductions of TCA-precipitable ^32^P up to 10.2%. As the pipetting error in this assay was determined to be 4% we assume that the nuclease activities in the capsid preparations were not significant.

The ^32^P capsid preparations were analyzed by Nycodenz gradient centrifugation. Nycodenz was chosen as it conserves protein interactions better than any other gradient media, allowing the recovery of functionally active protein complexes [32]. In our hands, Nycodenz allowed the recovery of 95% of *E. coli*-expressed capsids loaded, while CsCl, which is known to allow separation of genome-containing and empty capsids - caused significant capsid disintegration to 10% (data not shown). As Nycodenz has properties similar to sucrose that allows a separation based on the sedimentation coefficient. In contrast to sucrose however, Nycodenz allows capsids to reach their equilibrium. In order to determine sedimentation of unassembled core proteins, we analyzed urea disintegrated capsids by centrifugation. Due to the unphysiological disulfide bonds of the C terminal Cys upon expression in *E. coli*, the assay was performed using the C183S P-rC mutant.

**Figure 2:**
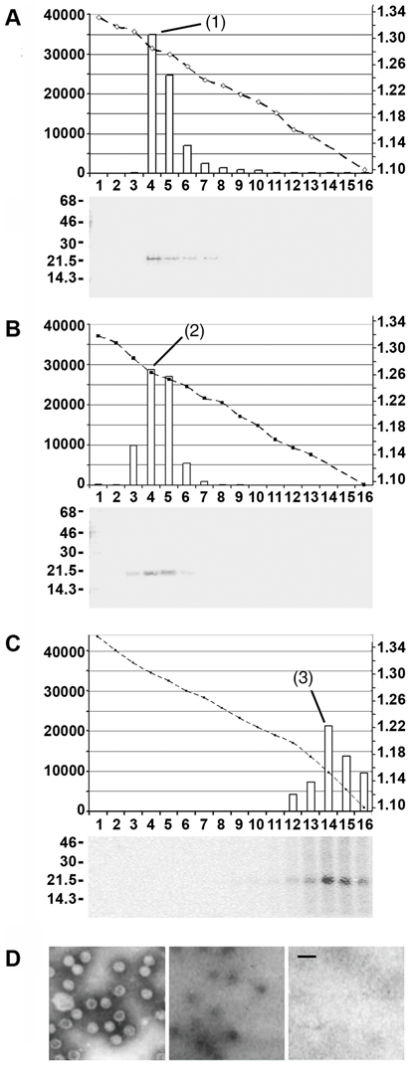
Analysis of capsids by Nycodenz gradient ultracentrifugation and electron microcopy. A–C: Nycodenz gradient ultracentrifugation using 50 ng of capsids. The graphs show the fractions (numbers below), their densities (right scales [g/ml] and dotted lines) and the radioactivity of the core protein bands based on phosphoimaging (left scales, arb. units). The autoradiographies below depict the radiography after SDS PAGE. A. P-rC, B. P-Mat-C. C. P-rC183S treated with 4 M urea prior to centrifugation. The figures demonstrate that P-rC exhibited a peak at 1.283 g/ml (1) while P-Mat-C showed a peak at 1.263 (2). core proteins from urea treated C183S P-rC migrated with a peak density of 1.156 g/ml (3). D. Electron micrograph of P-rC. Left panel: staining by phosphotungstic acid, middle panel: after incubation with Nycodenz, right panel: no stain. Note that contrast on the two right panels was enhanced as Nycodenz gives a faint contrast only. The bar represents 50 nm. The figure shows that Nycodenz diffuses into the capsids giving contrast within capsids lumen.

Sedimenting the capsids on Nycodenz gradient resulted in a peak of P-rC from 1.257 to 1.283 g/ml, with a maximum at 1.283 g/ml (Fig. 2A, peak (1)). The three major fractions contained 91% of the radioactivity. P-Mat-C banded at a slightly lower density between 1.242 and 1.284 g/ml with a maximum at 1.263 g/ml (Fig. 2B, peak (2)). The four major fractions contained 98% of the radioactivity indicating that capsids had not undergone significant degradation during centrifugation.

Urea dissociated core proteins remained close to the top of the gradient exhibiting a peak at 1.156 g/ml (Fig. 2C, peak (3)). Likely, these core proteins represent dimers as it was reported upon urea disintegration by others [30].

Despite the fact that rcDNA has a ca. 1.8 fold higher molecular mass than RNA, the density of P-Mat-C was slightly lower. In Nycodenz however, RNA has a higher density (1.18 g/ml) than dsDNA (1.13 g/ml) while proteins have 1.31 g/ml in Nycodenz [32]). Therefore, the different density distribution of Mat-C and rC implies that the gradient medium entered capsid lumen. To test this hypothesis we searched for Nycodenz entry by electron microscopy. Nycodenz, an electron-rich solute, can be seen without addition stain. Fig. 2D depicts that Nycodenz caused 20–24 nm large dots which were absent in the negative control. The size of the stain was similar to the lumen of P-rC stained by phospho tungstic acid, which exhibited the external diameter of 35 nm known for HBV capsids.

### Antibody-binding to capsids and capsid subassemblies

We used rC capsids in order to separate capsids from core protein dimers and capsid subassemblies. Separation was performed by Superdex 75 size exclusion chromatography using C183S rC capsids.

**Figure 3:**
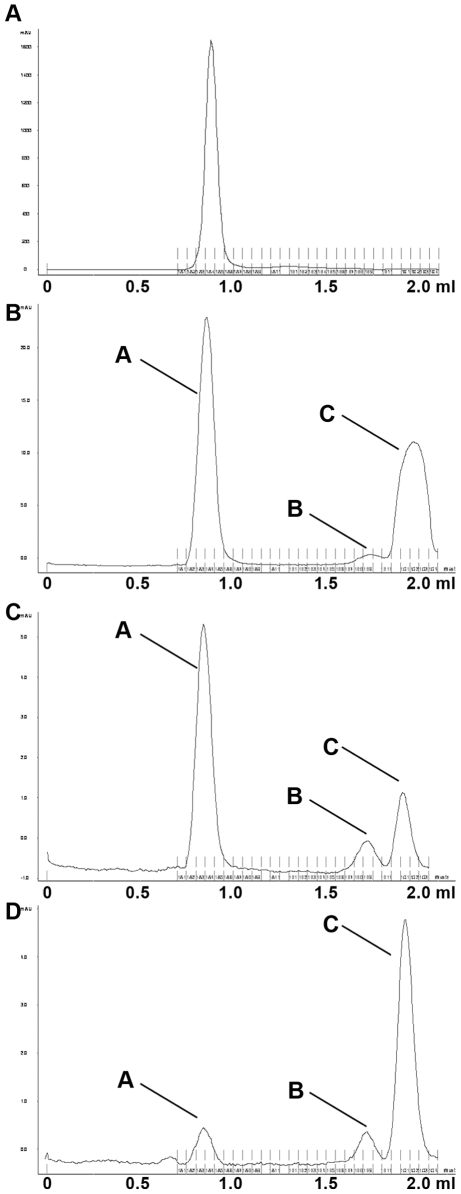
Equilibrium of capsids and capsid subassemblies. Separation of C183S rC subassemblies on Superdex 75 PC 3.2/30 columns. A. Separation of the stock with 100 µg/50 µl, B. stock of 10 µg/50 µl. C. Reinjection of fraction A3 (peak A from Fig. 3B) after 1 h at RT. D. Reinjection of fraction C1 (peak C from 3B) after 90 min at 4°C. The x-axes depict the fractions, the y-axes the OD_280_. The panels support the idea that capsids breathe, showing the capsid (peak A) and two capsid subassemblies (peaks B and C). Denaturation of capsids resulted in core proteins eluting in the flow through.

Separating a stock solution of C183S-rC revealed a single peak in fractions A4 (0.85 ml) (Fig. 3A). Its appearance in the exclusion volume of the column implies that practically all core proteins were assembled to capsids. However, when a 20 fold lower core protein amount was applied three peaks occurred (Fig. 3B): the strongest peak (peak A; 51% of total protein) appeared in fraction A3/4 (0.85 ml), which is within the exclusion limit of the column. A small peak (B, ∼3%) appeared in fraction B9 (1.73 ml) and another stronger peak (peak C, 46%) was observed in fraction C1/2 (1.92 ml).

Re-application of peak A after 1 h at RT resulted in the re-appearance of peaks A, B and C (69% peak A, 10% peak B and 23% peak C; Fig. 3C). Re-injection of peak C revealed the same peaks but with a different distribution (A 11%, B 10%, C 79%) (Fig. 3D). This finding confirms that rC undergoes dissociation, as described previously [1],[5].

For analyzing the immune reactivity of the fractions, we used two anti capsid antibodies: (1) polyclonal DAKO Ab reacts with entire wt capsids but only weakly with denaturated core proteins (<1% [18]), (2) monoclonal FAb3105 which was shown previously to bind to an epitope on core protein dimers involving the immune dominant loop (aa 77–80 and 83–84 [33]).

**Figure 4:**
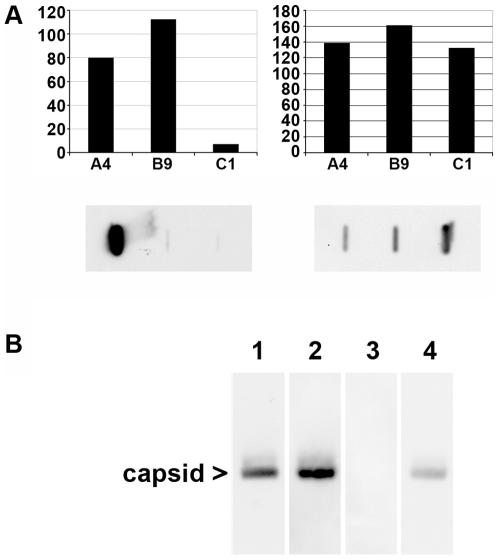
Characterization of anti capsid antibodies using capsid subassemblies. A. Immune reactivity of capsids and subassemblies using DAKO Ab (left panel) and FAb3105 (right panel). The graphs show the reactivity normalized to the protein amount (arbitrary units). The fractions are given below the x-axes. The reactivity on the y-axes is depicted in arbitrary units. The panels below are dot blots using peaks A-C from Fig. 3B(DAKO Ab) or from Fig. 3D(Fab3105). The graphs show that the polyclonal DAKO Ab reacts with capsids and fraction B9, while the FAb3105 reacted with all fractions equally well. B. Interference between DAKO Ab and FAb3105 binding to rC separation on native agarose gel electrophoresis. 1. 1^st^ Ab: DAKO Ab, 2^nd^ Ab: horse radish peroxidase-labelled (POD) anti rabbit. 2. 1^st^ Ab: FAb3105, 2^nd^ Ab: POD anti mouse. 3. 1^st^ Ab: DAKO Ab followed by FAb3105, 2^nd^ Ab: POD anti mouse. 4. 1^st^ Ab FAb3105 followed by DAKO Ab, 2^nd^ Ab: POD anti rabbit. The blots show that preincubation with DAKO Ab totally prevented capsid interaction with FAb3105 while preincubation with FAb3105 inhibited DAKO Ab reactivity significantly but not completely.

Determination of the immune reactivity of peaks A, B and C by dot blot is shown on Fig. 4A. The graph shows the reactivity, normalized to the protein amounts. FAb3105 reacted similarly well with all fractions giving evidence that they contained core proteins which were at least assembled to dimers. The polyclonal DAKO Ab reacted best with fraction B and somewhat less with fraction A containing the capsid. It must be considered however that the capsid peak contains the encapsidated heterogeneous *E. coli* RNA, which leads to an overestimation of the core protein amount. Reactivity with fraction C was faint. Given that peak C contained core proteins already assembled to dimers we conclude that the DAKO Ab recognizes preferably epitopes that are formed on capsid subassemblies with a higher complexity.

The interference of spike insertions with DAKO Ab binding implies binding at or close to the spikes, similar to FAb3105. We thus analyzed the antibody competition for their epitopes on rC. Fig. 4B shows that preincubation with DAKO Ab completely inhibited FAb3105 binding, suggesting overlapping binding sites. Saturation with FAb3105 prior to DAKO Ab incubation reduced DAKO Ab binding but not entirely, potentially due to its polyclonality.

### Nuclear import changes the density of Mat-C

In order to analyze the fate of Mat-C upon nuclear entry we analyzed the sedimentation in Nycodenz after subjecting P-Mat-C to nuclear import. Import reaction was performed using digitonin-permeabilized cells, which is a well-established system for analysis of nucleo-cytoplasmic traveling and HBV genome release at the nuclear envelope [19],[34]. A control reaction was performed by addition of WGA, which blocks active nuclear import by nuclear transport receptors [35]. Following import reaction, nuclei were lysed by the same non-ionic detergents used for capsid purification of Mat-C from secreted virus. A similar protocol was chosen to exclude that nuclear lysis has an impact on the subsequent capsid analysis on Nycodenz gradients.

**Figure 5:**
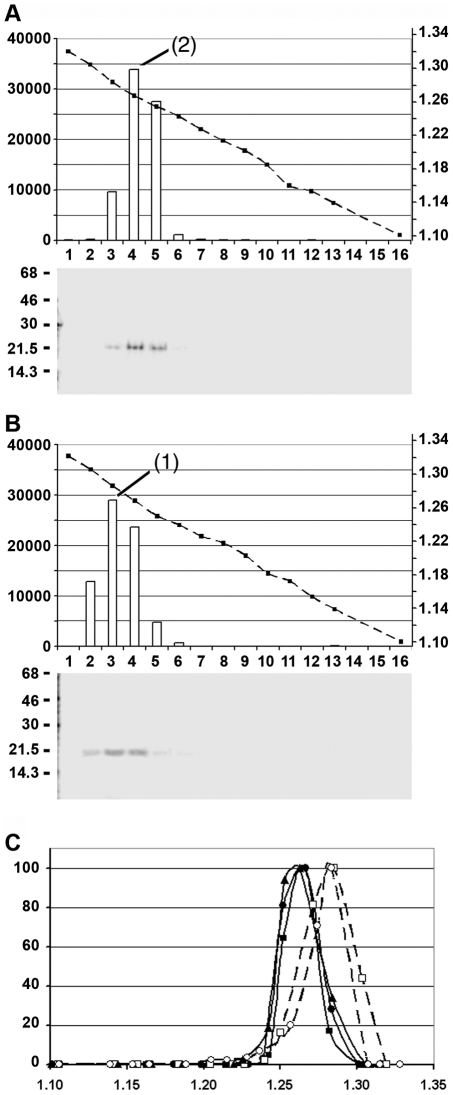
Migration of Mat-C after *in vitro* nuclear import. A. P-Mat-C after transport using 4×10^6^ Digitonin-permeabilized HuH-7 cells. B. as in the upper panels but nuclear import was blocked using WGA. Centrifugation and analysis as in Fig. 2A–C. The figures show that after nuclear transport the capsids migrated with a peak density of 1.285 g/ml (1) identical to the density of P-rC (Fig. 2A. Upon inhibition of transport by WGA, the capsids exhibited a peak at 1.267 g/ml (1), showing no difference with P-Mat-C (Fig. 2B, peak (2)). C. Superposition of densities and capsid migration on Nycodenz gradients. The y axis was normalized to depict the strongest signal as 100%. X axis: density in g/ml. Bold lines: P-Mat-C (full triangles), P-Mat-C from RNase A-treated WGA-blocked cells (full squares), P-Mat-C from WGA blocked cells (full circles). Dotted lines: P-Mat-C from cells (open squares), P-rC (open circles).

Upon WGA inhibition, 97% of P-core proteins of the P-Mat-C migrated as purified P-Mat-C without cell exposure, exhibiting a peak between 1.283 and 1,252 g/ml (Fig. 5A, peak (2)) with a maximum at 1.267 g/ml. It has been shown that P-Mat-C from transport reaction is attached to the nuclear import receptors Importin α and β [18]. Thus, one might expect a higher density. However, precipitating with the DAKO Ab capsids that were preincubated with the cytosolic extract showed, by immune blot, that the number of coprecipitated import receptors is rather low (4 molecules per capsid, not shown). This accounts for an undetectable increase of protein mass of <13% only.

Core proteins derived from P-Mat-C following nuclear import peaked between 1.304 and 1.250 g/ml with a maximum at 1.285 g/ml (Fig. 5B, peak (1)). This maximum was identical to the one of the purified RNA-containing P-rC (1.283 g/ml). As 99% of ^32^P core proteins were found within these fractions we concluded that all core proteins were assembled into particles without exhibiting significant amounts of dimers or subassemblies. A summary of all capsid sedimentation profiles is given in Fig. 5C. The requirement for an active nuclear import suggested however that the transition from “light” to “heavy” capsids occurred inside the nucleus.

### Effect of RNA on ^32^P-core proteins derived from nuclear import

**Figure 6:**
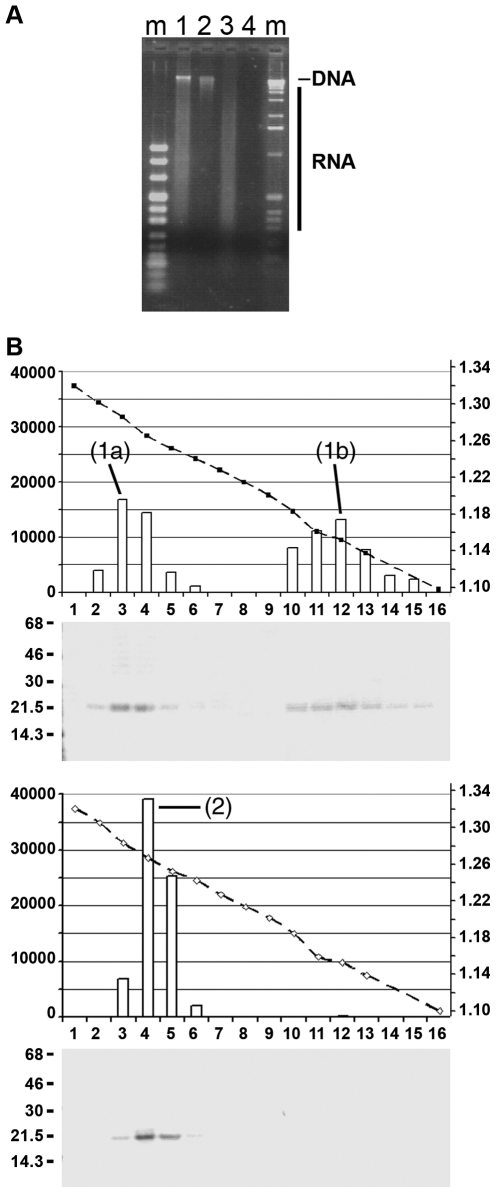
Effect of RNase A treatment of Digitonin-permeabilized cells on Mat-C migration. A. Control of RNase A treatment by ethidium bromide staining after agarose gel electrophoresis. m: marker, 1: cells not treated with nucleases, 2: RNase A-treated cells, 3: DNase I-treated, 4: RNase A and DNase I-treated cells. The Figure shows that RNase A-treatment degraded the cellular RNA completely. B. Effect of RNase A-treatment on P-Mat-C migration on Nycodenz gradients. Upper panels: RNase A-treated cells, lower panels: as in the upper panels but upon inhibition of nuclear import by WGA. For legend see Fig. 2A–C. The figures show that RNase A-treatment results in two peaks at 1.285 g/ml (1a) and 1.156 g/ml (1b). The low density peak exhibited the same density as urea disintegrated core proteins (Fig. 1B (3)). The high density peak showed a similar migration as Mat-C after nuclear import with the same peak density but with a broader range. When import was blocked P-Mat-C showed a migration with a peak at 1.266 g/ml (2) as P-Mat-C that was not exposed to cells (Fig. 1B peak (2)).

The changed density of the DNA-filled P-Mat-C to the density of RNA-filled *E. coli*-expressed capsids upon nuclear transport implied that the DNA genome in Mat-C was replaced by RNA. Such a replacement likely involves at least partial capsid disintegration, although transitory capsid subassemblies could not be detected in our assay. Reduction of temperature or modification of salt concentration - successfully used in biophysical assays [1] - could not be applied as physiological nuclear import is temperature and salt dependent. We used an alternative approach based on the observation that HBV capsid assembly rate is enhanced by core protein RNA-interaction [36]. We treated digitonin-permeabilized cells by RNase A, which is a small 13.7 kDa protein that is far below the threshold of diffusion into the nucleus (up to 68 kDa [37]). RNA degradation was monitored by ethidium bromide staining after agarose gel electrophoresis of the cell lysate (Fig. 6A). Specificity of degradation was shown by control digestions using DNase I (37 kDa). DNase I treatment however causes collapse of the nuclear structure with diffuse distribution of the nuclear pores (not shown).

RNase A-treated cells were used for nuclear import of P-Mat-C. Applying the nuclear lysate onto Nycodenz gradient showed two peaks, one with densities from 1.241 to 1.300 g/ml, with a maximum at 1.285 g/ml (Fig. 6B, peak (1a)) and a second with densities from 1.141 to 1.185 g/ml, with a maximum at 1.156 g/ml (Fig. 6B, peak (1b)). Quantification showed that 47% of the ^32^P core protein migrated in the first peak while 53% were found in the lighter fractions. The heavier peak showed the same peak density of P-Mat-C after nuclear import but with a broader distribution. The lighter one showed the same distribution as urea treated core proteins.

A control was performed by adding WGA during the import reaction. Here, core proteins migrated with the density of P-Mat-C, ranging from 1.242 to 1.282 g/ml (maximum 1.266 g/ml; Fig. 4B, peak (2)), showing that RNase A has no impact on P-Mat-C density prior to nuclear import.

### Analysis of P-Mat-C using size exclusion chromatography

**Figure 7:**
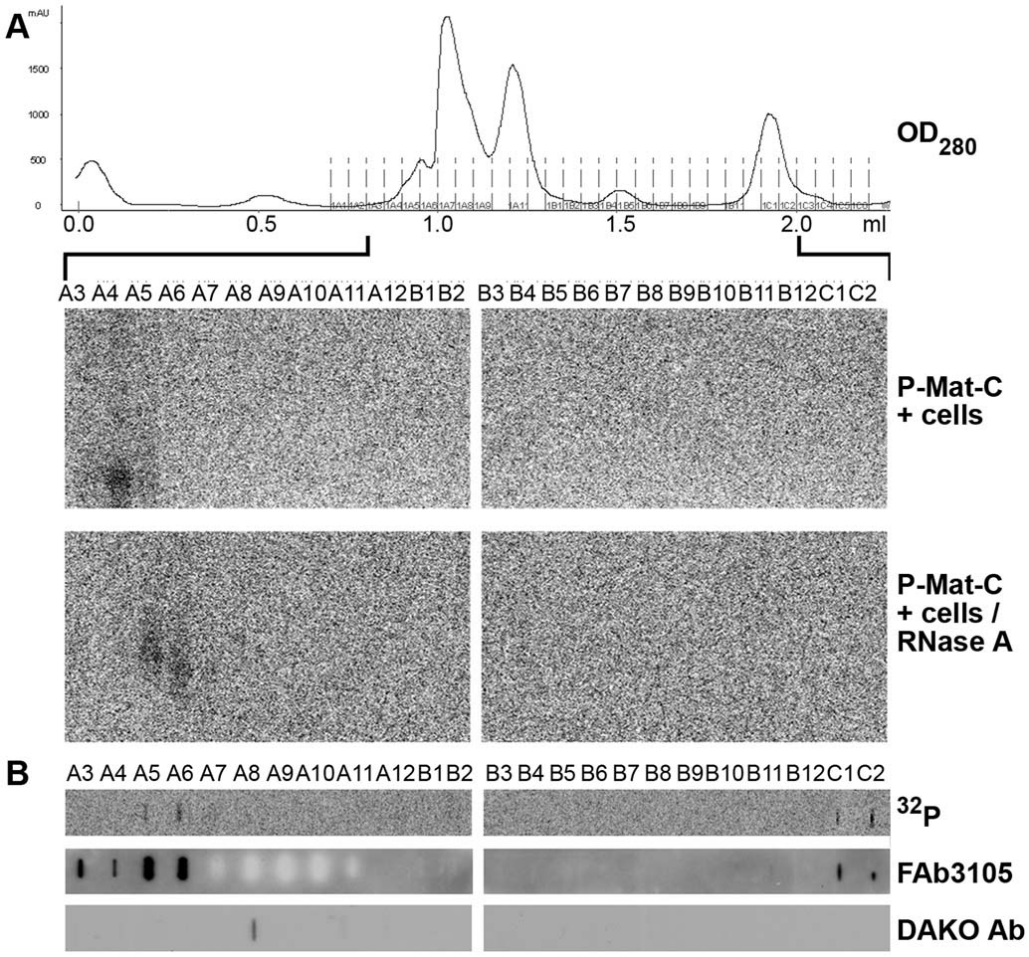
Analysis of Mat-C after nuclear import by Superdex 75 columns and native agarose gel electrophoresis. A. Upper panel: OD_280_ of the elution profile. The profile remained unchanged when cells were treated with RNase A. Middle panels: phosphoimager scan after native agarose gel electrophoresis. Lower panel: phosphoimager scan after native agarose gel electrophoresis using fractions from P-Mat-C added to RNase A-treated cells. B. Analysis of P-Mat-C from RNase A-treated cells by dot blot. Upper panel: phosphoimager scan, middle panel: detection by FAb3105, lower panel: detection by DAKO Ab.

To confirm the presence of capsids, dimers and capsid subassemblies, we analyzed capsids derived from nuclear import by size exclusion chromatography. Fig. 7A (upper panel) shows the elution profile at OD_280_. The peaks were derived from cellular proteins, as the same profiles were obtained from permeabilized cells without P-Mat-C and from RNase A-treated cells. We analyzed the presence of ^32^P capsids by native agarose gel electrophoresis (Fig. 7A, lower panels). In lysate from cells not treated with RNase A, ^32^P signals were obtained in fractions A3–5 migrating as integer rC capsids. ^32^P-core proteins from RNase A-treated cells, in contrast, were observed in fractions A5–7 and exhibited a migration slower and more diffuse than rC. No core proteins were observed in fractions B9 and C2 in which dimers and more complex capsid subassemblies were found. It must be considered however, that in native agarose gel electrophoresis unassembled core proteins migrate diffusely. Given that the signals were already at the detection limit, diffuse bands could have caused false negative results.

We thus analyzed the fractions derived from the RNase A-treated cells by dot blot (Fig. 7B, upper row), revealing ^32^P label in fractions C1/2 by phosphoimaging. Immune reaction of the dot blot by FAb3105 confirmed the presence of core proteins in all the ^32^P-positive fractions (Fig. 7B, middle panels). These fractions however were not reactive with DAKO Ab, which showed a faint signal only in fraction A8. As it overlaps with the peaks of cellular proteins, we assume that it results from unspecific interactions, which is in accordance with the absence of any FAb3105 or ^32^P label in this fraction.

### Analysis of light and heavy fractions from Nycodenz gradient centrifugation by anti capsid antibodies

**Figure 8:**
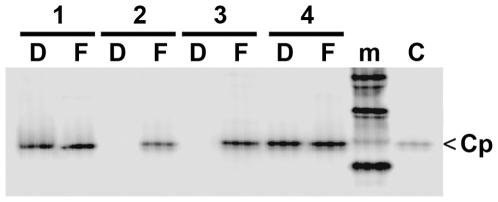
Immune precipitation of light and heavy capsid fractions from Nycodenz gradients after nuclear import of Mat-C. Phosphoimager scan after SDS PAGE. D: precipitation by DAKO Ab, F: precipitation by FAb3105, m: marker, C: P-Mat-C. 1: fractions 2–5 of P-Mat-C subjected to nuclear transport (1.250–1.304 g/ml), 2: fractions 2–5 from P-Mat-C added to RNase A-treated cells (1.241 to 1.300 g/ml), 3: light fractions of the same gradient (1.141–1.185 g/ml), 4: fractions 3–5, derived from RNase A-treated cells in which nuclear import was blocked by WGA (1.242 to 1.282 g/ml). m: marker, C: core proteins. The scan shows that FAb3105 precipitated core proteins from light and heavy fractions. DAKO Ab, in contrast, precipitated core proteins from heavy fractions in which capsids peaked, but not from heavy or light fraction of RNase A-treated cells.

To confirm the immune reactivity of the capsids, dimers and other capsid subassemblies, we investigated the light and the heavy fractions derived from Nycodenz gradient centrifugation by immune precipitation. Heavy fractions (2–5) from P-Mat-C exposed to untreated cells (1.250–1.304 g/ml) and heavy fractions 3–5, derived from RNase A-treated cells in which nuclear import was blocked by WGA (1.242 to 1.282 g/ml) served as controls. From the RNase A-treated sample, we analyzed the heavy fractions 2–5 (1.241 to 1.300 g/ml) and the light fractions of the same gradient (1.141–1.185 g/ml). Fig. 8 depicts that FAb3105 precipitated the ^32^P-core proteins in all heavy and light fractions from all gradients with similar efficiency. In contrast, DAKO Ab precipitated ^32^P-core proteins only from heavy fractions of P-Mat-C from untreated cells and from RNase A-treated cells in which nuclear import was blocked by WGA. No precipitate was found in the heavy and light fractions from RNase A-treated cells in which nuclear import occurred.

### Intranuclear immune fluorescence of capsids and subassemblies generated during capsid disassembly

Gradient analyses and size exclusion chromatography indicated that P-Mat-C is subject to an RNA-dependent intranuclear reassembly process upon nuclear import. For confirmation we analyzed the intranuclear appearance of capsids and their subassemblies in individual cells. Due to the lack of a suitable infection system we used Digitonin-permeabilized cells, which are a wide-spread technique for studying nuclear import including HBV capsids [18],[19],[34]. Digitonin permeabilizes the plasma membrane leaving the nuclear and ER membrane integer. After addition of exogenous cargos and the addition of nuclear import receptors the fate of the cargo can be analyzed using microscopy. We added Mat-C and rabbit reticulocyte lysate, which is common source of transport receptors [34]. The localization of capsids and capsid subassemblies was determined by confocal laser scan microscopy using indirect immune fluorescence. A control stain was performed with propidium iodide (PI) in order to depict cell nuclei.

**Figure 9:**
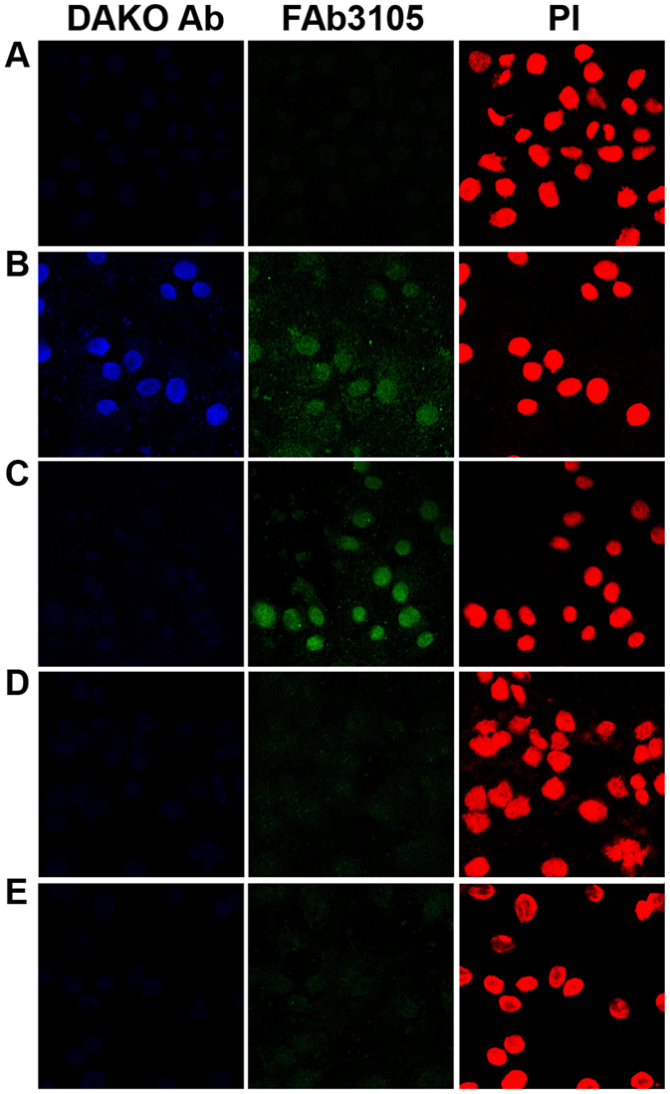
Intranuclear detection of capsids and their subassemblies after addition of Mat-C to Digitonin-permeabilized HuH-7 cells. Indirect immune fluorescence using DAKO Ab (blue) and FAb3105 (green). To indicate the nuclei a co staining by propidium iodide (PI) was performed (red). A: negative control without capsids, B: addition of Mat-C, C: addition of Mat-C but on RNase A-pretreated cells, D: as in B but upon inhibition of nuclear import by WGA, E: as in C but upon WGA addition. The figure shows that upon nuclear entry in untreated cells both DAKO Ab and FAb3105 reacted with nuclear Mat-C derived capsids. RNase A-treatment in contrast allowed detection of capsid subassemblies generated during assembly-disassembly processes by FAb3105 but not by the DAKO Ab.

Mat-C were added to permeabilized cells that were either untreated or RNase A-treated. Controls were performed by inhibiting nuclear import using WGA and by using cells to which no capsids were added. Fig. 9 shows that both DAKO Ab and FAb3105 exhibited nuclear fluorescence after addition of Mat-C to permeabilized cells which had not been treated with RNase A. This staining pattern is in accordance with data reported previously in permeabilized and integer cells [17],[19]. It is also in agreement with the typical findings in liver histology [38]. The signals of both antibodies were specific, as no fluorescences were observed in cells to which no Mat-C was added, or in which nuclear import was blocked by WGA [18]. In RNase A-treated cells however, no immune fluorescence could be observed using DAKO Ab, but strong signals were obtained using FAb3105. These findings are in accordance with the observation that capsid subassemblies present in the lysates from RNase A-treated cells were not recognized by DAKO Ab but recognized by FAb3105.

FAb3105 staining in RNase A-treated cells was significantly stronger than in cells untreated with RNase A. As both antibodies were added together this observation is in agreement with the competition for binding to capsids.

## Discussion

### Characterization of the anti capsid antibodies

Several studies describe the *in vitro* assembly of viral capsids, but there are only few investigations targeting their disassembly or their intracellular fate. *In vivo* investigations can hardly yield capsids in amounts suitable for biochemical analysis. We thus used two anti capsid antibodies and characterized at first their binding specificity for capsid subassemblies. Both antibodies, raised against entire capsids, are conformation-dependent. We used the well-studied FAb3105 in order to comparatively characterize the DAKO Ab for its reactivity against different capsid subassemblies, obtained by separation on size exclusion columns. Comparison was required, as the calibration of the column with globular proteins (see Material and Methods) showed that the migration of the subassemblies did not correspond to the MW of a single core protein (21.5 kDa) neither to a core protein multimer, so that a form-dependent retardation of the core proteins or unspecific interactions with the matrix were assumed. Both subassemblies (B, C) reacted with the FAb3105 and thus represented dimers or larger multimeric association of core proteins. Based on the known kinetics of HBV capsid assembly, we assume that peak C exhibits core protein dimers and peak B corresponds to a larger assembly state. Our experiments do not allow drawing a conclusion on how many dimers form this complex. According to the literature trimers of the dimers could be present in peak B, as this assembly state was shown to be the only distinct capsid subassembly population apart of dimers and capsids [10]. The DAKO Ab reacted with the capsid subassembly in peak B but poorly with the dimer fraction (C). In fact, the faint signal obtained after blotting of this fraction can be well explained by the limited formation of capsid subassemblies larger than dimers occurring between harvest from the column and blotting. We assume that the DAKO Ab either reacts with epitopes at the interface of dimers or, alternatively, the larger subassemblies may exhibit conformational differences compared to free dimers, as it was suggested previously [1].

The antibodies competed for their substrate implying that they both attach at, or near, the capsid spikes. This was not surprising, as the loop comprises the immune dominant c/e1 epitope of the capsids. In summary, the antibody characterizations indicate that DAKO Ab requires core protein assemblies larger than dimers, and that both antibodies exhibited sterical interference. Considering the kinetics of *in vitro* assembly [10], it must be assumed that the DAKO Ab reacts with core protein hexamers and fully assembled capsids.

### Distribution of capsids in density gradients

Anti capsid antibodies do not detect small amounts of core protein monomers. For following the migration of all states of capsids assembly on gradient we depended on sensitive and quantitative detection. We took advantage of capsid phosphorylation, which allows labelling of the core proteins by radioactivity. Phosphorylation of the capsids did not significantly affect capsid structure, as indicated by their unaltered migration on native agarose gel electrophoresis and their unchanged reactivity with the DAKO Ab (not shown). This result was expected as the number of transferred phosphates was low. In addition, data from others have shown that *E. coli*-expressed capsids are identical to liver-expressed capsids within a resolution limit of 30 Å [39]. Recently these data were confirmed with better resolution of 16 Å further showing that no gross structural changes are linked with genome maturation and envelopment [40]. Better resolution with 10 Å however showed that a hydrophobic pocket is present on DNA-containing capsids [41].

As these differences do not affect capsids size, the different densities of Mat-C and rC in Nycodenz gradients suggest that the nucleic acid content caused the difference. This assumption is in accordance with the reported densities of RNA and dsDNA in Nycodenz [32]. Entry of Nycodenz in the capsid lumen was proven by electron microscopy, and could have occurred either via the 1.5 nm-measuring holes in the capsid shell [12], or by capsid breathing.

### Effect of nuclear transport on P-Mat-C sedimentation

For analyses of the intracellular fate of HBV capsids, we investigated the dissociation reaction of HBV capsids using digitonin-permeabilized cells. Addition of P-Mat-C to permeabilized cells caused a change of migration towards the density of RNA containing capsids, but only upon active nuclear import. Although the differences appear to be minor, the superposition of the different gradients allows a clear differentiation of the sedimentation profiles (Fig. 3B).

Strikingly, P-Mat-C showed a single density peak after nuclear import, implying that virtually all capsids were converted, and that no significant subpopulations failed to deliver their encapsidated DNA into the nucleus. This high efficiency in our system suggests that it may reflect the situation in infected individuals where up to 80% of all HBV particles are infectious [20].

### Effect of nuclear RNA on nuclear capsids and capsid subassemblies

Genome release and subsequent replacement of the genome by RNA could hardly be explained by passage through holes in the threefold or quasi-threefold axis of the capsid shell [12]. It can be concluded from structural data on HBV capsids that even dissociation of one core protein hexamer upon capsid breathing would cause holes of only 4.3 – 5.7 nm. Further dissociation of the capsid is probably necessary for genome release. The required incubation period of the transport assay for obtaining detectable nuclear import (15 min) was however much longer than the short concentration-dependent association times of >50 s observed *in vitro* [29].

To observe the disassembly of capsids into subassemblies within nuclei we decelerated reassociation by removal of RNA. Both sedimentation and size exclusion chromatography showed two reaction products. The smaller product corresponded in size to urea treated C183S P-rC by sedimentation, and to peak C of the chromatography, thus likely representing core protein dimers. This assumption was confirmed by their reactivity with FAb3105 and their failure to bind to the DAKO Ab following both methods of separation.

The second product migrated similarly to RNA filled capsids, but exhibited a slightly broader peak. Size chromatography confirmed a similar but nevertheless different migration than capsids, as the peak was shifted by two fractions. Further confirmation was obtained by native agarose gel electrophoresis, after chromatography, showing a diffuse and retarded migration compared to capsids. Such a migration was reported for P-rC upon acid denaturation [18] causing protein aggregation. Immune blotting confirmed the absence of DAKO Ab reactivity arguing against capsid or core protein hexamer formation similar to that observed after acid denaturation. The remaining reactivity with FAb3105 indicated however core protein dimer formation, so that we conclude that the oligomeric reaction product consists of core protein dimers which were assembled in a misfolded manner. In fact, misdirected folding under preservation of intact dimer formation was observed recently upon addition of the HBV capsid assembly inhibitor HAP1 [42].

### Localization of assembly/disassembly

To confirm the intranuclear localization of the dissociation and reassociation events, we analyzed capsids and their subassemblies by indirect immune fluorescence in permeabilized cells. In accordance with gradient centrifugation and size exclusion chromatography, we observed that RNase A-treatment generated intranuclear subassemblies, which were reacting with FAb3105 but not with the DAKO Ab. Despite the absence of capsids, the nuclear presence of core protein dimers indicates that RNase A treatment did not interfere with nuclear import of core protein. This supports the conclusion that HBV capsids become imported into the nuclear basket as entire particles [43] but disassemble in the nuclear environment.

In the presence of RNA, in contrast, DAKO Ab and FAb3105-reactive assembly forms of the core protein appeared. Considering the absence of detectable capsid subassemblies between complete capsids and core protein dimers upon *in vitro* disassembly [30], this finding implies that the capsids disintegrate to core protein dimers followed by a rapid RNA-dependent reassociation to capsids, which is misdirected in the absence of RNA. As we used unphosphorylated Mat-C in this assay, these results further exclude that phosphorylation of Mat-C has had a significant impact on formation of capsids and on the capsid subassemblies.

### Conclusions

**Figure 10:**
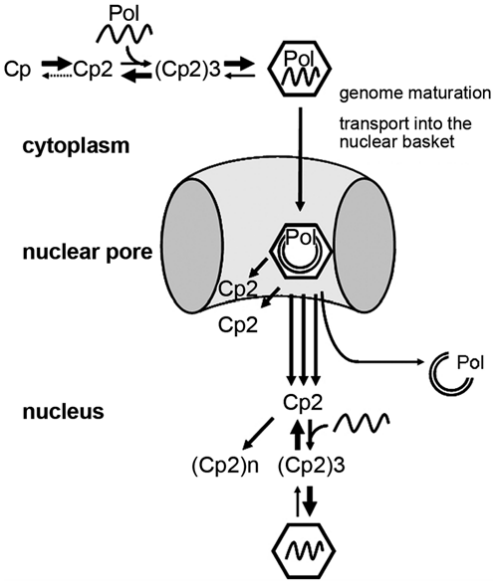
Schematic drawing of localization-dependent assembly and disassembly of HBV capsids. Cp2: core protein dimers, (Cp2)3: defined capsid subassemblies, (Cp2)n: core protein polymers, Pol: polymerase, icosahedra: capsid, waved line: RNA, circles: DNA, ellipses: section of a nuclear pore complex. For details see text.

In the present paper we showed that the dissociation of the HBV capsid follows the *in vitro* association reaction in inverse direction. Our observation that core proteins reassemble to capsids inside the nucleus implies that both compartments - the cytoplasm in which initial capsid formation occurs - and the nucleus, support the assembly reaction. The environment in which disassembly occurs should be consequently before capsid entry into the free karyoplasm. A potential candidate compartment would be the nuclear basket at the karyoplasmic side of the NPC, as it provides the unique proteins of the NPC. We thus hypothesize that the capsids assemble from core proteins via dimer and hexamer formation, as it was proposed recently [10],[29] (Fig. 10). The time at which the polymerase-RNA pregenome complex interacts with the capsid subassemblies has to be left open but may enhance the assembly reaction. During genome maturation and transport, the capsid is subject to capsid breathing and remains stable [17],[19],[43]. Apparently, the basket of the nuclear pore comprises the environment promoting capsid disintegration and genome liberation, which occurs after arrest of the capsid [19],[43]. We assume that core protein dimers derived from disassembled capsids are however able to diffuse deeper into the nucleus. When the threshold concentration of nuclear core protein dimers [36] is reached, capsid formation would occur, which is probably enhanced by cellular RNA. As cytoplasmic capsids, they undergo breathing but remain stable, explaining the capsid accumulation observed in liver biopsies.

## Materials and Methods

### Preparation of capsids

Mat-C were prepared and purified from virions of the permanently Hepatitis B virion-expressing hepatoma cell line HepG2.2.15 [44] accordingly to Rabe et al [19]. The capsids of these virions, which were shown to be infectious in chimpanzees [45], contain viral DNA in a relaxed circular form. [44]. *E. coli*-derived capsids (rC) and a mutant in which the C terminal Cys was replaced by Ser (C183S rC) were expressed and prepared as described previously [12]. Electron microscopy did not show any difference between wt capsids and this mutant (not shown). While Mat-C showed strong contamination of proteins of cell culture medium (approx. 30 fold) both *E. coli-*expressed capsid preparations exhibited high purity of >90% with respect to the total protein: SYPRO staining after SDS PAGE showed a single band of 21.5 kDa when 400 ng were loaded. Disintegration of capsids was achieved by treating C183S rC by 4 M urea for 10 min at 42°C.

### Nuclease activities in the capsid preparation

All capsid preparations were analyzed for nuclease contaminations by incubating 50 ng capsids with ^32^P labelled nucleic acids in 50 µl transport buffer for 2 hours at 37°C in siliconized Eppendorf tubes and determining the amount of radioactive TCA precipitable material at 0 and 120 min. Five µl were removed and spotted in duplicates on Whatman 3 M filters, dried, then immersed in a beaker containing 300 ml 5% TCA and 1% PPi for 15 min on ice. Filters were rinsed 3 times 5 minutes with 300 ml 5% TCA 1% PPi, then immerged 1 minute in 70% ethanol, dried and finally counted in a liquid scintillation counter. Radiolabelled DNA was obtained by random priming of a 700 nt PCR product, whereas radiolabelled RNA was obtained by T7 transcription of a linearized plasmid containing a cloned gene downstream a T7 promoter. Radiolabelled DNA and RNA were separated from free unincorporated nucleotide either by spin column or by ethanol precipitation in the presence of ammonium acetate. Single stranded DNA was obtained by 5 min heat denaturation of DNA at 100°C followed by rapid chilling in ice.

### ^32^P labelling of capsids

Mat-C was labelled by addition of [γ^32^P]ATP using the activity of the *in vivo* incorporated cellular protein kinase (P-Mat-C) as described previously [19] and resulted in the transfer of 2–4 P/capsid (T4) which corresponds to 0.008–0.017 P/core protein P. rC and C183S rC were labelled *in vitro* by PKC according to Kann et al. [26] (P-rC and P-C183S). In brief this phosphorylation requires partial disintegration of capsid structure by low salt treatment, followed by phosphorylation. Capsids from the stock solution were at first diluted 1∶20 in water and incubated for 15 min at 40°C. 5 µg of the diluted capsids were preincubated in PKC buffer (20 mM HEPES pH 7.4; 10 mM MgCl_2_; 1.7 mM CaCl_2_; 1 mM DTT) together with 3 µg phosphatidylserine for 10 min at 42°C. Then 15 ng protein kinase C (Promega) and 10 µCi [γ^32^P]ATP (3000 Ci/mmol) were added (5 µl final volume) and the kinase reaction let to proceed for 30 min at 37°C, after which 0.5 µl PBS 10X was added. In contrast to the previously reported protocol we did not performed an RNase A digest, resulting in a 50fold reduced labelling of about 0.01 P/core protein. After phosphorylation the capsids were reconstituted by adjusting the salt concentration to physiological concentrations. As we have shown previously by EM these capsids exhibit the same structure than original capsids [26].

For separation by native agarose gel electrophoresis, capsids were loaded on the gel using sample buffer without SDS. Electrophoresis was performed on 1% agarose/TAE using a TAE buffer. Blot onto PVDF membranes was performed according to Southern [46]. Quantification of radioactivity was performed by phosphoimaging using either Typhoon 9200 (Amersham Biosciences) or Pharos FX (BioRad). Quantification of immune blots was performed by ECL (PerkinElmer) using ChemiDoc XRS (BioRad).

### Transport assays using digitonin-permeabilized cells

For analysis of capsid integrity during transport on Nycodenz gradients, or on Superdex 75 PC 3.2/30 columns, 4×10^6^ HuH-7 cells were permeabilized by digitonin as described previously [19], with the modification that the permeabilized cells were harvested after the washing steps prior to the transport reaction. The transport was performed as described, but using a volume of 100 µl comprising 50 ng P-Mat-C and rabbit reticulocyte lysate. If required, 100 µg/ml of wheat germ agglutinin (WGA) was added during the washing steps. After incubation, cells were lysed using 1% NP-40/PBS/5 mM MgCl_2_ for 1 h at 37°C. After lysis, Triton X-100 was added to a final concentration of 0.2%, followed by 10 min incubation at 37°C and subsequent sonification (6×15 sec). The sample of 200 µl was then subjected to analysis. RNase A-treatment was performed for 15 min at 37°C at a final concentration of 4 µg/µl.

Transport assays for immune detection, immune staining and subsequent confocal laser scan microscopy were done accordingly to Rabe et al. [19]. Immune staining was performed with the polyclonal rabbit anti capsid antibody (1∶200, DAKO Ab) and with the monoclonal mouse anti capsid protein antibody (1∶200, Fab3105, Institute of Immunology CO., LTD, Tokyo, Japan). Cy5-labelled anti rabbit antibody (1∶400, Dianova) and FITC-labelled anti mouse antibody (1∶200, Dianova) served as secondary antibodies. Nuclei were stained with propidium iodide (1∶5000).

### Nycodenz gradient centrifugation

Two hundred µl of samples were added onto 3.6 ml continuous Nycodenz/TN gradient (1.11–1.32 g/ml) in a SW60 rotor. Centrifugation was done for 22 h at 10°C and 36,000 rpm. Fractions of 220 µl were harvested. Density d was determined by refractometry (σ) using the formula: d = (σ×3,287) – 3,383. The fractions were vortexed prior to density determination.

### Size-exclusion chromatography

Capsid preparation was centrifuged for 5 min at 17 000 g before being processed through the size-exclusion column. The apparent molecular size of the proteins was analyzed by chromatography on a Superdex 75 PC 3.2/30 column (GE Healthcare), which has a fractionation range of 3 to 70 kDa. The column was equilibrated with transport buffer (20 mM HEPES pH 7.3, 2 mM Magnesium acetate, 110 mM Potassium acetate, 5 mM Sodium acetate, 1 mM EGTA and 1 mM DTT). Proteins (50 µl) were eluted with a flow rate of 40 µl/min and recorded by continuously monitoring the absorbance at 280 nm. The column was calibrated with the following standard proteins: ovalbumin (43 kDa; 1.13 ml)), chymotrypsinogen A (25 kDa; 1.26 ml), RNase A (13.7 kDa; 1.36 ml) and the void volume was determined with dextran blue (>2000 kDa).

### Immune blot and immune precipitations

Quantification of the capsids were done by immune blot accordingly to Rabe et al. (11) using either the polyclonal rabbit anti capsid antibody (1∶5000, DAKO Ab) or the monoclonal mouse anti capsid protein antibody (1∶2500, Fab3105). As secondary antibody, a horse radish peroxidase anti rabbit or anti mouse antibody (1∶10000, Dianova) were used. Detection was performed by ECL (PerkinElmer) using ChemiDoc XRS (BioRad). For immune precipitations 3.5×10^6^ sheep anti rabbit- or anti mouse-conjugated biomagnetic beads (Dynal) were added to 22 µg DAKO Ab or mouse anti capsid protein antibody (Fab3105) and incubated in 0.1% BSA/PBS overnight at 4° C on a rotating wheel. Afterwards unbound antibodies were removed by washing the beads three times with 0.1% BSA/PBS. The antibody saturated beads were subjected to light and heavy capsid fractions from Nycodenz gradients in the presence of 0.1% BSA and incubated overnight at 37°C on a rotating wheel. The precipitate was washed three times in 0.1% BSA/PBS, one time in 0.1% Nonidet P-40/PBS, transferred into a new cup and again washed three times with PBS. The samples were loaded onto a 4–12% Bis-Tris gradient gel SDS-PAGE (NuPAGE) and transferred onto a PVDF (VWR International) by electro blotting. Precipitated capsid proteins were detected by their intrinsic radioactive signals using phosphoimaging.
